# Supplementary material for: Early patterning followed by tissue growth establishes distal identity in Drosophila Malpighian tubules
Source: Front Cell Dev Biol. 2022 Aug 19;10:947376. doi: 10.3389/fcell.2022.947376 (PMC9437309; doi:10.3389/fcell.2022.947376)
Supplement: Supplementary file 1 [file Image1.pdf]

Supplementary material

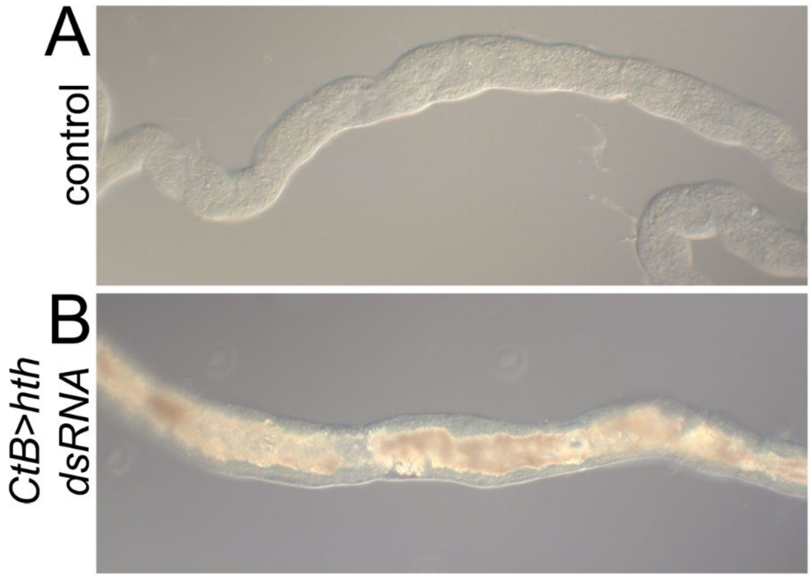

Figure S1. Excessive uric acid accumulates in adult Malpighian tubules following *homothorax* knockdown. (A) Section of a control (OrR) adult MpT. **(B)** Section of a *hth* RNAi (*CtB>hth dsRNA*) adult MpT.

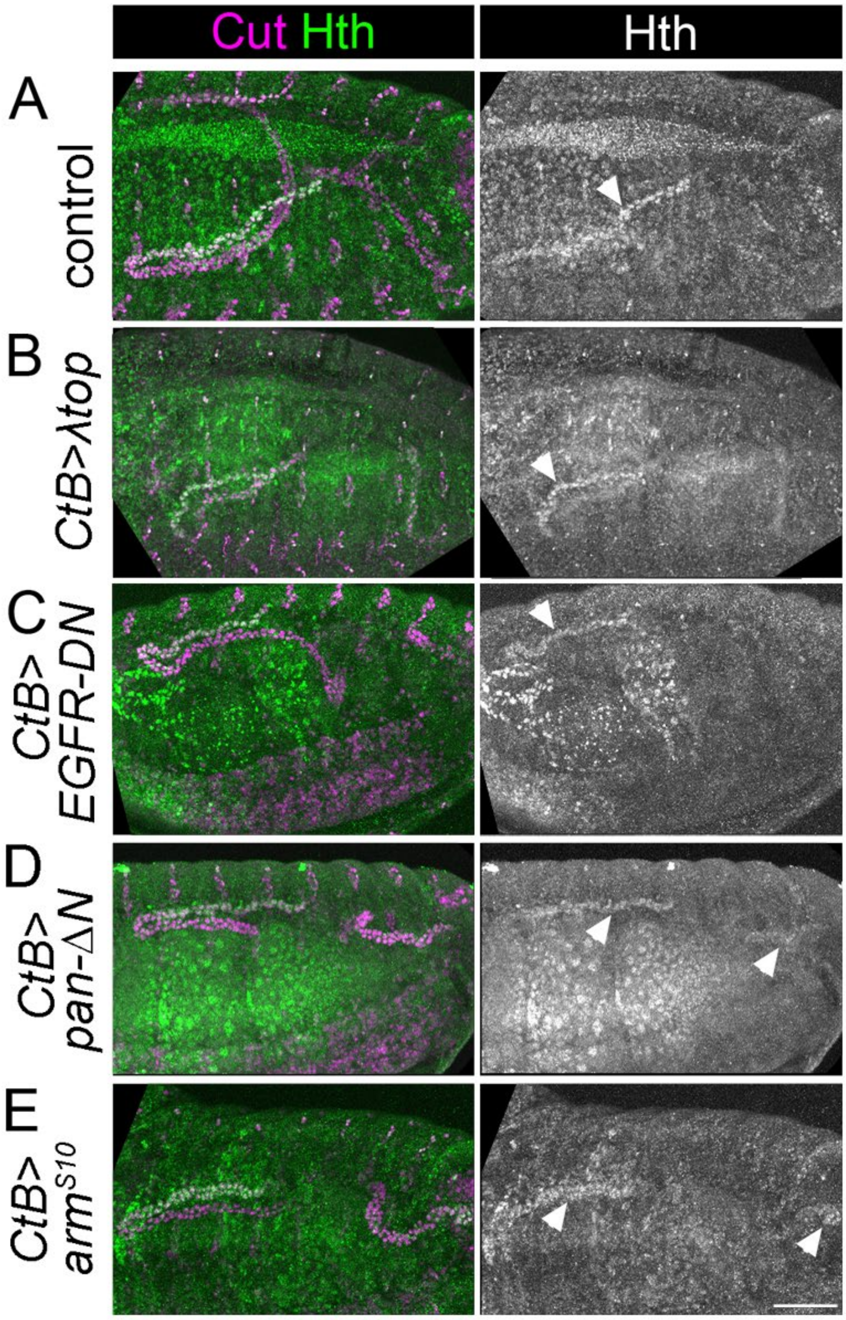

Figure S2. EGF or Wg pathway activity do not appear to pattern the distal Malpighian tubule. (**A-E**) ~Stage 16 embryos stained to show MpT nuclei (anti-Cut) and the nuclei of cells from the distal MpT segment (anti-Hth, arrowheads). In each example at least one of the pair of anterior MpTs is clearly visible. **(A)** Control (*w*^{1118}) embryo. **(B)** *CtB-Gal4>UAS-λtop* embryo. **(C)** *CtB-Gal4>UAS-EGFR-DN* embryo. **(D)** *CtB-Gal4>UAS-pan/dTCFΔN* embryo. **(E)** *CtB>UAS-arm*^{S10} embryo. Scale bar = 50µm.

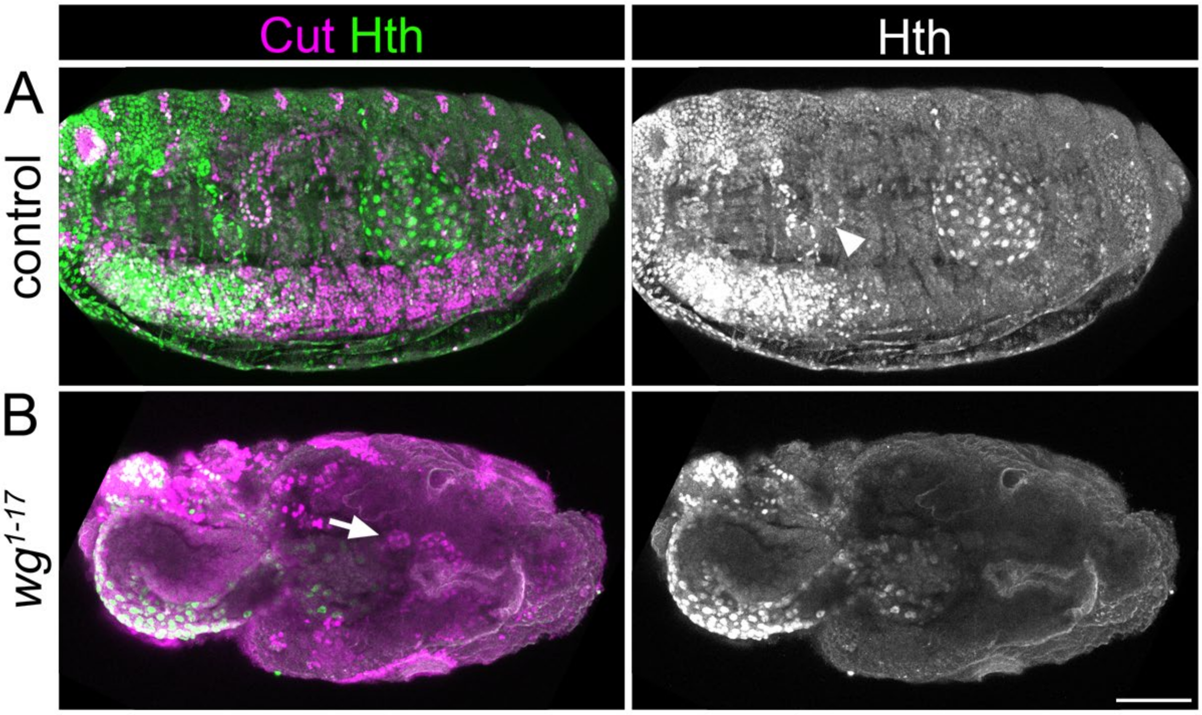

Figure S3. Wingless is required for expression of distal tubule markers. ~Stage 16 embryos stained to show MpT nuclei (anti-Cut) and the nuclei of cells from the distal MpT segment (anti-Hth, arrowhead). Arrow indicates MpT, where anti-Hth staining is absent. **(A)** Control embryo (*wg*$^{1\text{-}17}$/+ or +/+). **(B)** Homozygous *wg*$^{1\text{-}17}$ embryo. Scale bar = 50µm.

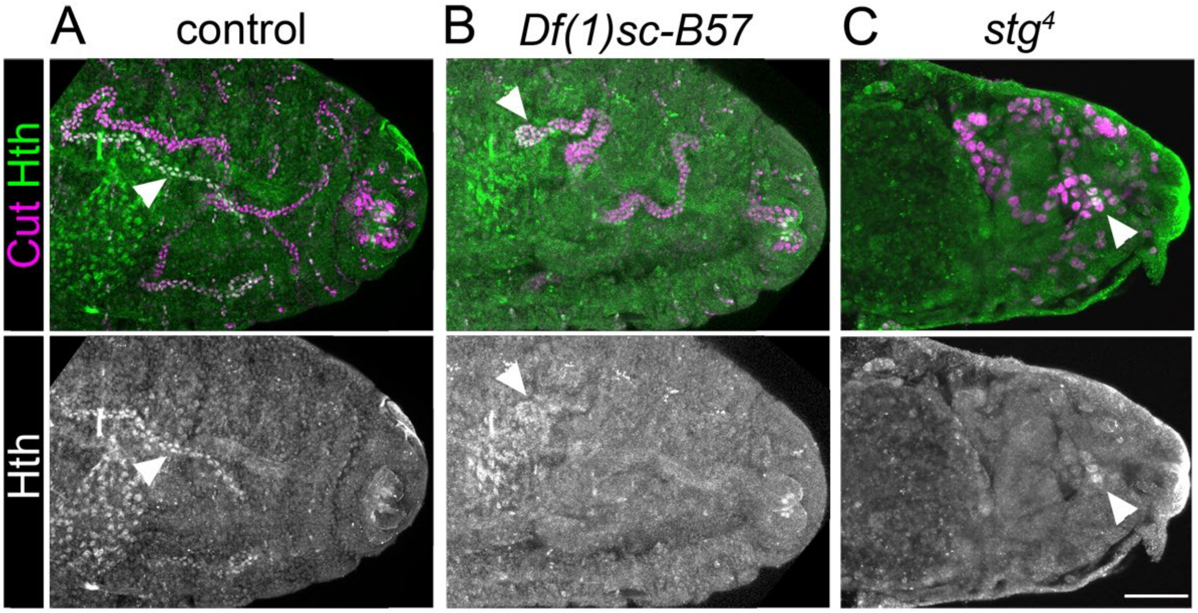

Figure S4. achaete-scute complex genes are not required to specify distal tubule identity, but are required to expand the pool of distal identity cells. ~Stage 16 embryos stained to show the nuclei of the MpT cells (anti-Cut) and nuclei from the distal MpT cells

(anti-Hth, arrowheads). **(A)** Control (*w*^{1118}) embryo (same embryo as Fig. 2D). **(B)** Homozygous *Df(1)sc-B57* embryo. **(C)** Homozygous stg^4 embryo. Scale bar = 50μm.

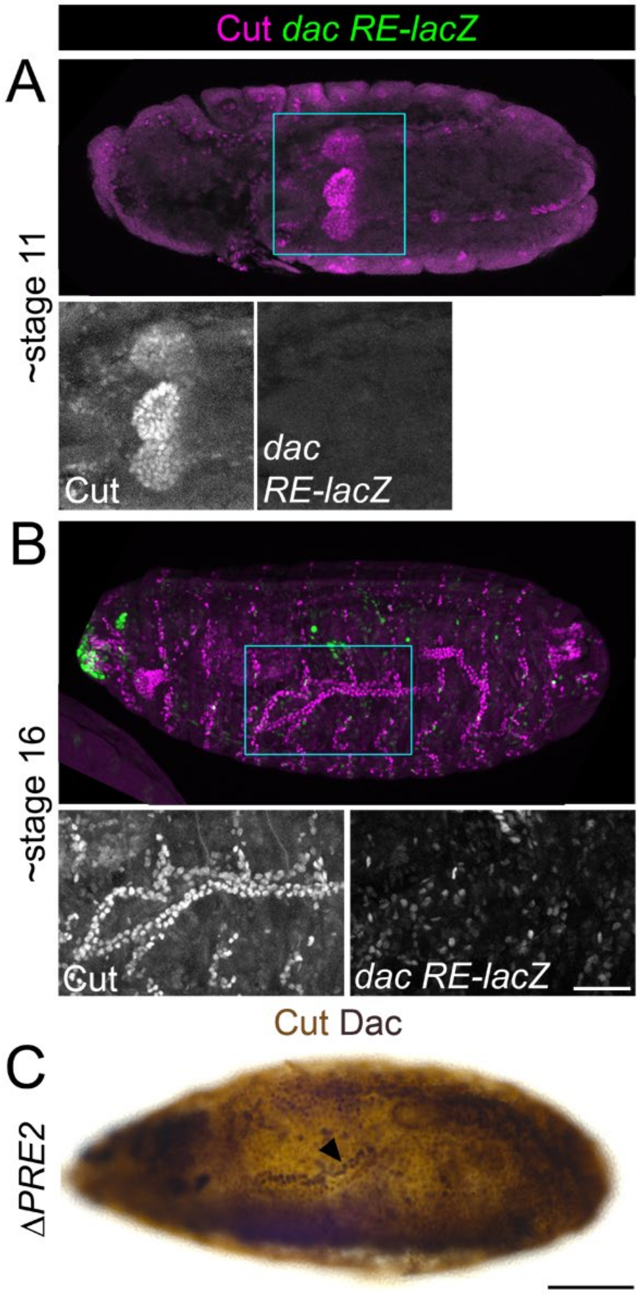

Figure S5. *dac RE* and *dac PRE2* enhancers do not regulate Dachshund expression in the developing Malpighian tubules. (A-B) *dac RE-lacZ* embryos stained with anti-β-galactosidase, along with anti-Cut to mark the MpT nuclei. This *dac* enhancer is characterised as driving expression in the leg disc, and contains putative dTCF/Pan binding sites (Giorgianni and Mann, 2011). Single channel images equate to region in blue box of merged image. **(A)** ~stage 11. **(B)** ~stage 16. (**C**) ~Stage 16 homozygous mutant embryo lacking the *PRE2* regulatory region of *dac* (Δ*PRE2*) stained to show MpT nuclei (anti-Cut)

and the nuclei of cells from the distal MpT segment (anti-Dac, arrowhead). *PRE2* is a Polycomb response element which has been characterised as a regulatory region for *dac* during leg development (Ogiyama et al., 2018). We were interested in this site as ChIP-on-chip from *Drosophila* embryos showed it to contain a dTCF/Pan binding site (Junion et al., 2012). Scale bar = 50μm.
